# Photometric Calibration and Image Stitching for a Large Field of View Multi-Camera System

**DOI:** 10.3390/s16040516

**Published:** 2016-04-11

**Authors:** Yu Lu, Keyi Wang, Gongshu Fan

**Affiliations:** Department of Precision Machinery & Precision Instrumentation, University of Science and Technology of China, Hefei 230022, China; lycn@mail.ustc.edu.cn (Y.L.); fgs1226@mail.ustc.edu.cn (G.F.)

**Keywords:** large field of view, multi-camera system, camera response function (CRF), lens vignetting model, dynamic range

## Abstract

A new compact large field of view (FOV) multi-camera system is introduced. The camera is based on seven tiny complementary metal-oxide-semiconductor sensor modules covering over 160° × 160° FOV. Although image stitching has been studied extensively, sensor and lens differences have not been considered in previous multi-camera devices. In this study, we have calibrated the photometric characteristics of the multi-camera device. Lenses were not mounted on the sensor in the process of radiometric response calibration to eliminate the influence of the focusing effect of uniform light from an integrating sphere. Linearity range of the radiometric response, non-linearity response characteristics, sensitivity, and dark current of the camera response function are presented. The R, G, and B channels have different responses for the same illuminance. Vignetting artifact patterns have been tested. The actual luminance of the object is retrieved by sensor calibration results, and is used to blend images to make panoramas reflect the objective luminance more objectively. This compensates for the limitation of stitching images that are more realistic only through the smoothing method. The dynamic range limitation of can be resolved by using multiple cameras that cover a large field of view instead of a single image sensor with a wide-angle lens. The dynamic range is expanded by 48-fold in this system. We can obtain seven images in one shot with this multi-camera system, at 13 frames per second.

## 1. Introduction

The omnidirectional camera is a type of ideal imaging device that has been increasingly used in recent years. The major advantage is its wide field of view (FOV), which allows it to capture a view of the entire scene. The synthesis of small patches into a large field of view has other advantages, such as reducing lens design difficulties. In addition, the dynamic range of a single sensor with a certain exposure setting has difficulties in meeting an extremely high dynamic range with wide FOV. The omnidirectional camera brings new prospects in various areas such as surveillance, tracking, visual navigation, localization and SLAM, structure from motion, photogrammetry, reconstruction of cultural heritage, and so on. A polycamera is a kind of non-central omnidirectional camera, comprising a cluster of conventional cameras pointing to different directions in a particular configuration. Recent attempts have been made to develop this type of camera. The PANOPTIC [1] is a hemispherical multiple camera system that uses 44 cameras covering 360° × 180° FOV. The AWARE [2] series camera consists of a multiscale optical layer, an optoelectronic readout layer, and an image presentation and interaction software layer. It uses hundreds of image sensors to address the conflict between FOV and resolution. The design methodology is described in detail in [3,4,5]. In [6], a cylindrical distributed polycamera was introduced based on FPGA hardware, using five digital cameras with wide-angle lens that provide a field of view with 360° horizontal azimuth.

These cameras can generate realistic panoramas for distant scenes. A polycamera is a kind of non-central omnidirectional camera; parallax exists between sub-cameras, and CRFs of the sensors are different. Low-pass filtering and smoothing methods are used in image blending for realistic visual effects. The simplest way to create a final composite is to simply take an average value at each pixel, but this method usually does not work effectively because of exposure difference, misregistrations, and scene movement. A better weight averaging method is the weight decrease from pixels near the center of the image to pixels near the edge [7]. Gain compensation and the multiband blending method [8,9] can effectively resolve exposure differences, misregistrations, and other factors. However, these methods do not consider the actual luminance of an object, but only achieve vision requirements.

In this study, a calibration method of the photometric characteristic of cameras is introduced. Dark current, radiometric response, and chromatic response difference of sensors of the large FOV multi-camera are measured accurately. Vignetting (decrease in intensity toward image edges) artifact patterns have been tested using an integrating sphere uniform light source. The actual luminance of an object is retrieved through results of obtained sensor calibration, and simultaneously removal of saturated pixels and correcting the nonlinear response of the sensor. The corrected image data are used in blending of images to make panoramas more objectively reflect the objective luminance. The calibration result not only enhances panorama realism but also is an essential step in photometric 3D reconstruction, structure from motion and photogrammetry.

The rest of the paper is structured as follows: Section 2 introduces the hardware of the multi-camera system with large field of view. Section 3 describes the photometric modeling and calibration method. Section 4 presents our calibration and image stitching results. In Section 5, we present conclusions and ideas for future work.

## 2. Overview of the Large FOV Multi-Camera System

A state-of-the-art CMOS sensor is used to build our camera system. Field Programmable Gate Array (FPGA) is used to set the exposure time of the sensors, acquire RAW image data, and transfer data to PC. The computer host is used to collect image RAW data, demosaic the Bayer image, restore the image, and process the data further. The multi-camera system is compact and flexible.

### 2.1. Sensor and Lens

The sensor module utilized in our system is an OmniVision OV3640, which supports quadruple extended graphic array (QXGA) resolution, *i.e.*, 2048 × 1536 and any size that scales down from QXGA, such as 1080 p and VGA [10]. Charge-coupled device (CCD) and CMOS detectors each have strengths and weaknesses coming from their architecture or their fabrication process [11,12]. With consumer digital camera equipment extensively used and semiconductor technology advances, the image quality of the CMOS image sensors has been greatly improved since they first appeared, and it provides a digital interface rather than an analog one. The Serial Camera Control Bus (SCCB) is a universal control protocol for the OmniVision image sensor and the details are described in [13]. It can be used to communicate between FPGA and OV3640 sensors, controlling data formats, gains, offsets, exposure times, and so on. The sensor module has a 3.1 mm focal length and a f/2.8 f-number, and a relatively wide field of view of 65°. Seven sensors are mounted on a 3D-printed structure. The optical axis angle between the central sensor and outer sensor is 40°. In addition, the outer sensors are evenly distributed along a circle.

### 2.2. Established Multi-Camera System

Hardware platform is needed for sensor exposure setting and image data acquisition, comprising central FPGA [14], DDR3 SDRAM frame cache, and camera link interface to transmit data. The data acquisition function is implemented in the central FPGA and involves four sub-blocks, which are sensor controllers, data transmit multiplexer, DDR3 memory controller, and camera link interface.

**Figure 1:**
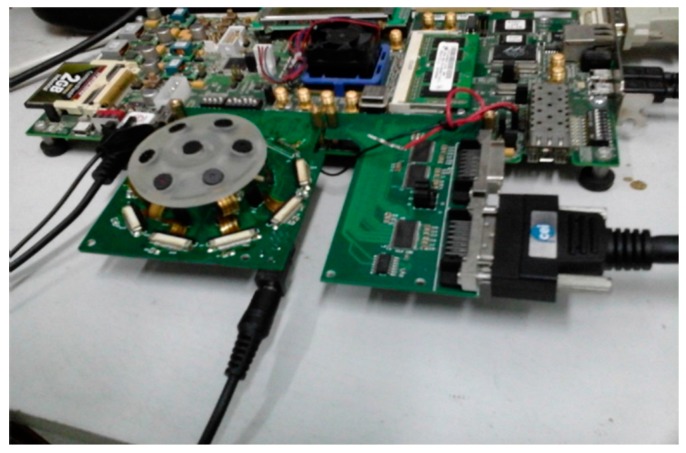
The multi-camera system adapted to shoot images with a large field of view.

The sensor controller module sets the exposure setting of each sensor, which are unique exposure times and other common parameters, namely, window, frame rate, and gain. The data cache includes two types, the line FIFO cache and the multiple frame DDR3 SDRAM cache. The image sensor outputs data line-by-line, and the line FIFO cache is used to match data rate between the image sensor and DDR3 memory controller, which are asynchronous modules. Exposure times are not equal; thus, shutters shoot at the same time but frames may not be output simultaneously. To obtain the fastest possible frame rate from the camera system, the slowest frame time or the longest exposure time is used as the base. Seven sensors shot at the same time, and the frame of the sensor with shorter exposure time is output earlier and then cached in DDR3 SDRAM; the frame of the sensor with longer exposure time is output later and cached in DDR3 SDRAM. Redundant faster frames are discarded during image frame with longer exposure time output. Multiple camera frames are cached in the same DDR3 SDRAM. The bandwidth of “full” camera link mode can reach 5.6 Gb/s. All data generated by the seven sensors are cached in DDR3 SDRAM, and then read out in accordance with the camera link specification. This multi-camera system can operate at 13 fps. The complete camera system, which includes seven sensors, a FPGA hardware platform, and camera link image data transfer interface, is shown in Figure 1.

## 3. Photometric Modeling and Calibration Method

A digital camera includes three parts, namely, the optical lens, image sensor, and electronic system. Vignetting of the lens decreases intensity toward image edges. The photosensitive material and manufacturing technique of the sensor determine the area of light sensitivity as well as its sensitivity level. Dark current, fixed pattern noise and shot noise are also different due to manufacturing processes. The R, G, B channels of the color sensor have different response characteristics. Different sensors also exhibit similar characteristics. Therefore, each channel of each sensor must be calibrated separately and then normalized in a radiometric calibration process. Opto-electronic converted charge must be amplified, quantized, and read out. Amplifier noise, quantization noise, and noise caused by data transmission line and PCB result in differences in obtained and actual values. A complete calibration of the sensor requires corrections in the domains mentioned, and is applied to measurement equation. Linear and nonlinear response of total dynamic range is a critical factor in the system, and is solved in the calibration process.

### 3.1. Vignetting and Radiometric Response Modeling

As described, a digital number of image pixels is determined by the input light energy, optical lens, opto-electronics converted charge, and electronic system. Vignetting is the cosine-4th law of illuminance falloff [15]:

(1) $$E^{\prime }=E_{0}^{\prime }\cos^{4}\omega^{\prime }$$

where ${E^{\prime }}_{0}$ is illuminance of on-axis, $E^{\prime }$ is illuminance of off-axis, and $\omega^{\prime }$ is off-axis angle. The off-axis illuminance attenuation caused by the cosine-4th effect of the imaging lens needs to be corrected prior to analyzing the data.

The multi-camera is used in visible light and the light from the integrating sphere is white. We don’t consider the wave length, while different color channels are take into consideration. L is illuminance of incident light(lux). Luminous power(lm) of sensor received can be expressed as:

(2) $$E_{i}=\frac{\pi k_{i}AL}{4}{\left(\frac{D}{f}\right)}^{2}$$

where A is the sensor area, $k_{i}$ is lens transmittance and it is constant for three channels (*i.e.*, $k_{r}$, $k_{g}$, *k_b_*), $\frac{D}{f}$ is the aperture. $R_{i}$ represents the channel response (electron/lm), T is the exposure time, so the generated analog signal intensity of pixel can be expressed as follows:

(3) $$V_{i}=TE_{i}R_{i}$$

After quantization, amplifier and transmission, we can obtain the final digital number (DN) of the image pixel is given by:

(4) $$DN_{i}=GV_{i}+DC+N_{s}+N_{R}+N_{Q}$$

where G is the gain of the sensor, DC is the number of electrons resulting from dark current, $N_{s}$ is the zero mean Poisson shot noise, and $N_{R}$ is the zero mean read noise generated by the amplifier. Quantization noise $N_{Q}$ is also shown to be zero mean, which is generated by analog-to-digital (ADC) conversion. Zero mean noise can be removed through averaging multiple samples [16,17,18].

The DN of the image pixel after averaging can be write as:

(5) $$DN_{i}=GTE_{i}R_{i}+DC$$

where DN and L may not show strict linearity. The obtained DN of image pixels can be fitted to illuminance by a polynomial equation [16]:

(6) $$DN=DC+P_{1}L+P_{2}L^{2}+P_{3}L^{3}+\cdots$$

The dynamic range of a sensor is limited by the sensitivity of the photosensitive material and the bits of photoelectron quantization when shooting a scene with a certain exposure time *t* and gain G. The dynamic range of the multi-camera system can be extended by shooting the same scene with multiple exposure settings. The pixel number range is from 0 to 255 with 8-bit quantization. The dynamic range can be extended by combining multiple sensors with different exposure time and gain. Suppose $EX_{\max}$ and $G_{\max}$ are the maximum exposure time and gain. $DC_{\max}$ is the dark current when the exposure time and gain are both maximized. $DC_{\min}$ is the dark current when the exposure time and gain are both their minimum values, that is, EX is 1-stop, and G is ×1. According to Equation (6), we can obtain the effective maximum illuminance of incident light $L_{\max}$ when both the exposure time gain is the minimum and the digital number DN is the maximum but not saturated. Identically, the effective minimum illuminance of incident light $L_{\min}$ can be solved when the both exposure time gain is the maximum and the digital number DN is minimum but above dark current $DC_{\max}$. The dynamic range(DR) can be expressed as:

(7) $$DR=L_{\max}/L_{\min}$$

### 3.2. Calibration Process

**Figure 2:**
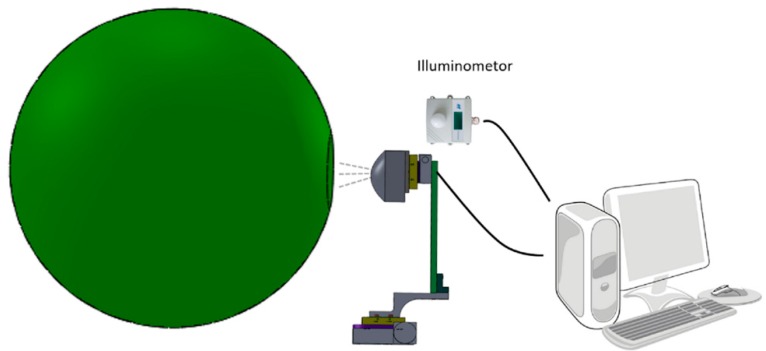
Photometric calibration with an integrating sphere.

In this section, we describe the calibration process. A complete calibration of the multi-camera system requires corrections in several domains to be applied to the model equations. According to the photometric model, the quantitative relationship between illuminance of incident light and digital value of image pixel can be determined in the laboratory. An integrating sphere was used as calibration light source for its advantages of large aperture and FOV, as well as uniform intensity [19]. The amount of light illuminance output can be changed by controlling the number of lighted LEDs, which are fixed in the integrating sphere. Wavelength of LEDs output are not considered in our calibration process. The illuminance is read from an illuminometer through an RS232 port at each exposure state. The test method of vignetting is the acquisition of images with an imaging lens under appropriate illuminance and analysis intensity distribution on the image. We also test the bare sensors (no imaging lens) illuminated by 13 LEDs for linearity and non-linearity response. In order to make camera with different point direction face the output port of the integrating sphere, the multi-camera system is mounted on a two-axis platform. Figure 2 shows the photometric calibration diagram.

For the color image sensor, the R, G, and B channels have different response exposures for the same illuminance. Based on the model of radiometric response, that is, Equation (6), the three channels of radiometric response are:

(8) $$\left\{ \begin{array}{l} DN_{r}=DC_{r}+P_{1r}L+P_{2r}L^{2}+P_{3r}L^{3}+\cdots +P_{nr}L^{n}\\ DN_{g}=DC_{g}+P_{1g}L+P_{2g}L^{2}+P_{3g}L^{3}+\cdots +P_{ng}L^{n}\\ DN_{b}=DC_{b}+P_{1b}L+P_{2b}L^{2}+P_{3b}L^{3}+\cdots +P_{nb}L^{n} \end{array}\right.$$

We need to collect sufficient images with different exposure state *DN_k_* (*k* = 1,2,3…,*n*), illuminance *L_k_* (*k* = 1,2,3…,*n*), and dark current *DC_k_* (*k* = 1,2,3…,*n*). By making *k* ≥ *n*, the total coefficients can be solved.

## 4. Calibration Results and Image Stitching

### 4.1. Lens Vignetting

**Figure 3:**
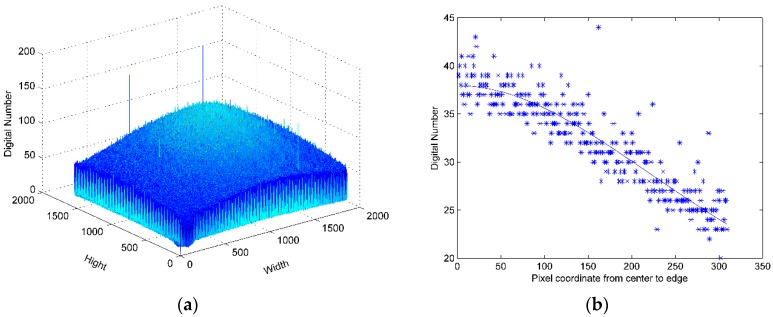
(**a**) Response distribution; (**b**) Distribution of red channel from center to edge and nonlinear least square fitting result.

**Figure 4:**
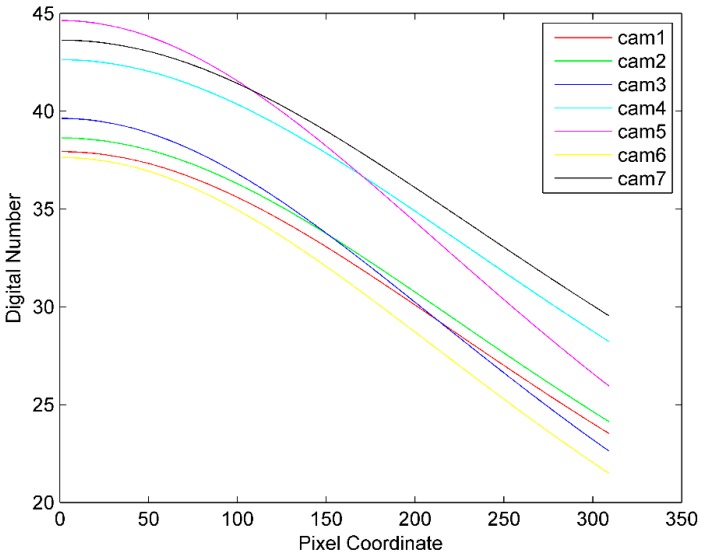
Vignetting mode of seven cameras.

We choose one direction of illuminance falloff mode as lens vignetting under the assumption the lens is centrosymmetric. The sensor response distribution is shown in Figure 3a with four lighted LEDs in the integrating sphere (22 lux), and the distribution of the red channel from center to edge is shown in Figure 3b. The vignetting mode of seven cameras is shown in Figure 4.

### 4.2. Dark Current

**Figure 5:**
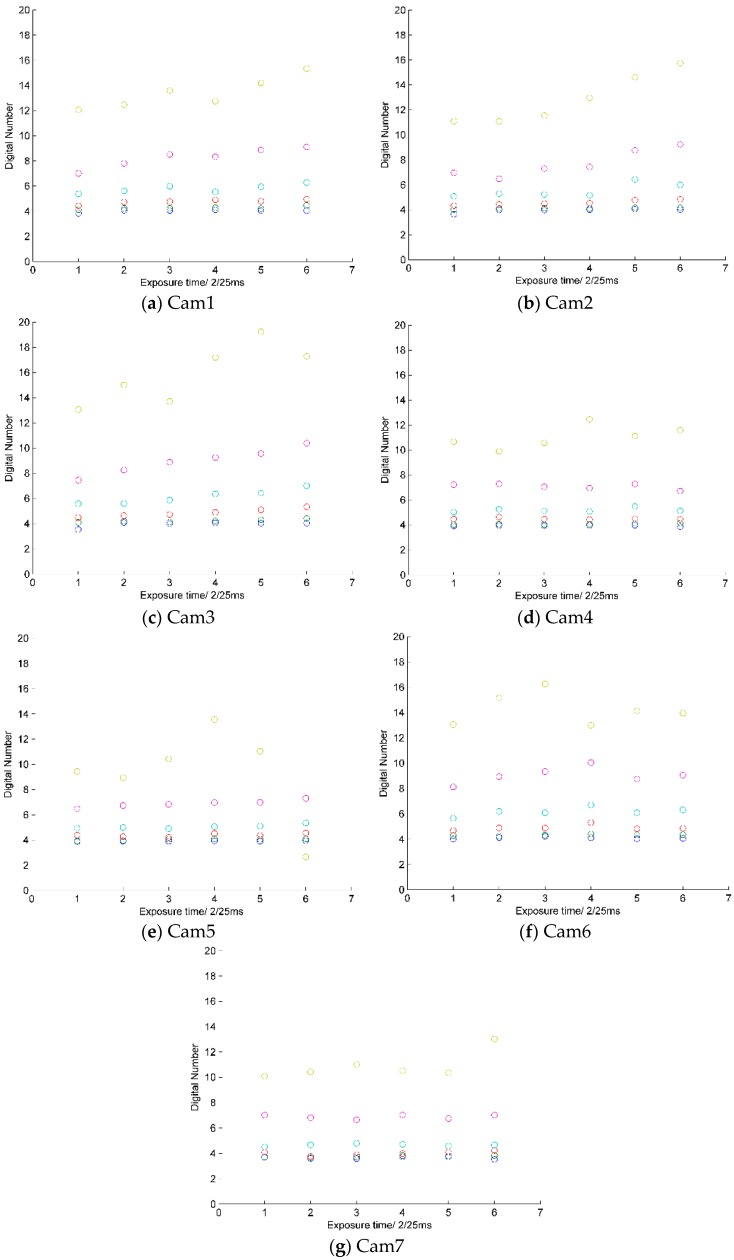
Dark current of seven sensors with six exposure times and six gain settings.

We tested dark current with six exposure time settings (2/25; 4/25; 6/25; 8/25; 10/25; and 12/25 ms) and six gain settings (×1; ×2; ×4; ×8; ×16; and ×32). In order to minimize the influence of thermal effects on the sensor output data, *i.e.*, dark current, we begin to store sample data after the bare sensor worked for 5–10 min in a dark room. The mean of the image channel pixels digital number is used as the final dark current number. The test results are presented in Figure 5. The results show that dark current increases significantly as the gain increases, but not with the exposure time. The dark currents of the R, G, B channels are almost at the same level, so we need not separate these channels for dark current in the subsequent calibration data process. The image data is 8-bit (or gray value between 0–255). The gains ×16 and ×32 are not used in real image data acquisition because these two gains result in uncertain dark current and low signal-to-noise ratio.

### 4.3. Radiometric Response

**Figure 6:**
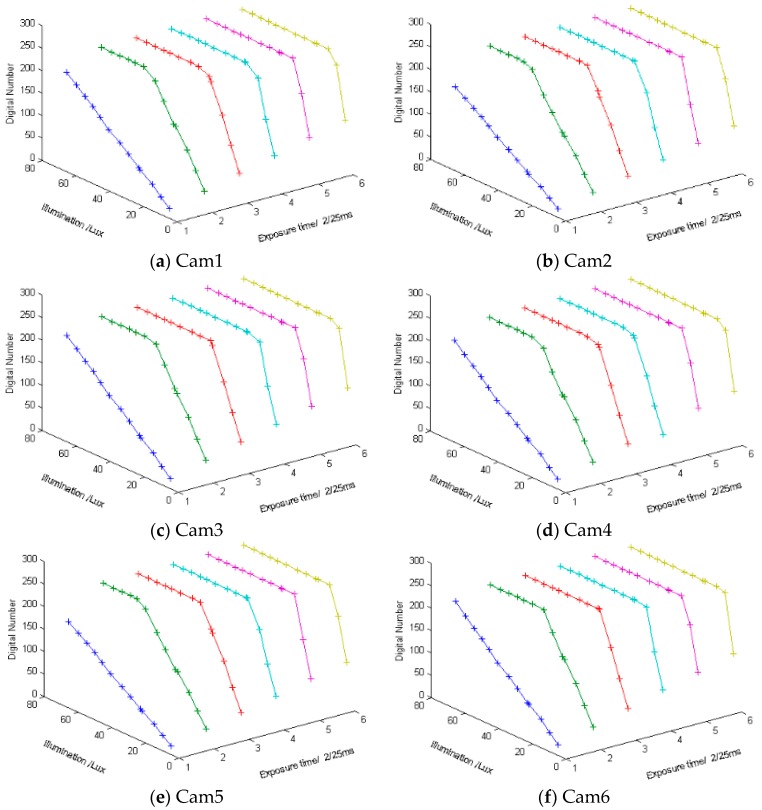

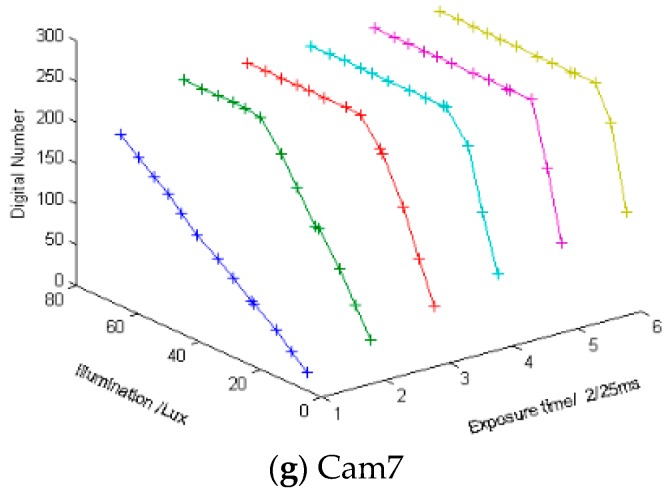
Red channel radiometric response of bear sensor with six exposure times (2/25 ms, 4/25 ms, 6/25 ms, 8/25 ms, 10/25 ms, and 12/25 ms), a digital gain ×1 and 0–80 lux illuminance. X-axis is exposure time, and the unit is 2/25 ms, Y-axis is illumination and the unit is lux, Z-axis is mean value of red channel digital numbers.

In order to calibrate the radiometric response of the sensors, we have tested the response with all settings which are all configurable exposure times and gains of sensors under different illuminances output by the integrating sphere. The exposure timesettings are 2/25 ms, 4/25 ms, 6/25 ms, 8/25 ms, 10/25 ms, and 12/25 ms. The gain settings are ×1, ×2, ×4, ×8. The illuminance output by the integrating sphere are between 0–80 lux. The red channel radiometric response results are shown in Figure 6. The figures show that the shorter the exposure time is, the greater the linear range is. The linear range is small with long exposure time, so the long exposure time setting is suitable for homogeneous illuminance. Different sub-cameras have different pixel digital number responses under the same illuminance. The longer the exposure time is, the greater the pixels digital number is. We have used Equation (8) as objective function to fit the sample data with linear fitting (*N* = 1) and four order polynomial fitting (*N* = 4). The results of Cam 1 are shown in Table 1.

The four-order polynomial fitting results and linear fitting results are almost equal as the sum of squares due to error (SSE) and the coefficent of determination (R-square) results show. The results show that P2, P3, P4 are too small, SSE of linear and four order fitting are very close and R-square almost equals 1. Thus we use the linear model as the radiometric response model. According to the fitting results, we can obtain illuminance range with linear response under a certain exposure setting. The non-linear response are the pixels which are under the level of dark current and above the start of saturation. When an illuminance output by the integrating sphere and a gain setting of sensor are unchanged and fitting the response values of increasing sensor’s exposure time step-by-step in the linear response range, the R-square of the linear fitting result is 1. The R-square of the exponential fitting result is also 1 when the gain setting is increased exponentially, *i.e.*, ×1; ×2; ×4; and ×8. Based on these radiometric responses corresponding with exposure time and gain setting, a proper exposure setting (*i.e.*, exposure time and gain) can be obtained quickly in certain circumstances. A reference is provided for the final image display and illuminance of projection from object to sensor is obtained.

DN/Lux represents the ratio of the digital number of pixels with incident light illuminance. The ratio value is from fitting results when the dark current is subtracted and pixel values are not saturated. The photosensitive material and manufacturing technique of the sensor determine the sensitivity level. The result shows that the order of sensitivity from high to low is blue, green, and red channels for the same illuminance. We can further calcutate the luminance of an object based on the sensor exposure setting and the quantitative relationship shown in Table 2.

The luminance quantization is only 8-bit when shooting a scene with a certain exposure setting. A visible image can be obtained shooting in an insufficiently lit environment when using long exposure time and large gain and a visible image can also be obtained shooing in a bright environment when using a short exposure time and little gain. The dynamic range can be extended by combining multiple sensors facing in different directions and with different exposure settings. We can roughly calculate that the minimum illuminance value with exposure setting one (*i.e.*, *Ex* is 6 stop and G is ×8) and the maximum illuminance value with exposure setting two (*i.e.*, *Ex* is 1 stop and G is ×1). The range of pixel values lies between *DC* to (255 – DC). We can know the illuminances under these two exposure settings when the pixel value are *DC* and 255 but not saturated. The dark current *DC_max_* (*EX* is 6 stop, and *G* is ×8) is almost twice as the *DC_min_* (*EX* is 1 stop, and *G* is ×1) as shown in the dark current calibration results. According to Equation (7), the dynamic range of the multi-camera system is almost 48 times as large as that of single sensor exposure at certain exposure times and gain settings.

### 4.4. Correcting Image and Stitching

We have collected seven images using the multi-camera system. The exposure times of Cam1 to Cam7 were 8/25 ms, 4/25 ms, 4/25 ms, 2/25 ms, 4/25 ms, 6/25 ms, and 12/25 ms, respectively. The gain settings are all ×1. We have subtracted the dark current and corrected vignetting, and then converted the pixel values to illuminance according to the camera photometric calibration results.

**Figure 7:**
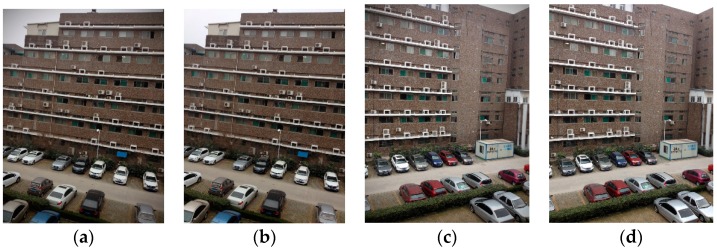
(**a**,**c**) Original images; (**b**,**d**) images with vignetting and photometrics corrected.

The perspective projection relationship between images can be estimated ahead because the cameras are fixed with a certain geometry and the optical axis angle between the central sensor and outer sensor is 40°. The multi-band blending method provided by Brown [9] is used to blend the stitched panorama image. Finally, the stitched image is projected on a sphere. The image output by Cam3 is shown in Figure 7a and the image output by Cam6 is shown in Figure 7c. The corrected images are shown in Figure 7b,d. The original images cannot accurately reflect photometric the brightness, such as the sky portion, while the corrected images are more photometrically compatible.

**Figure 8:**
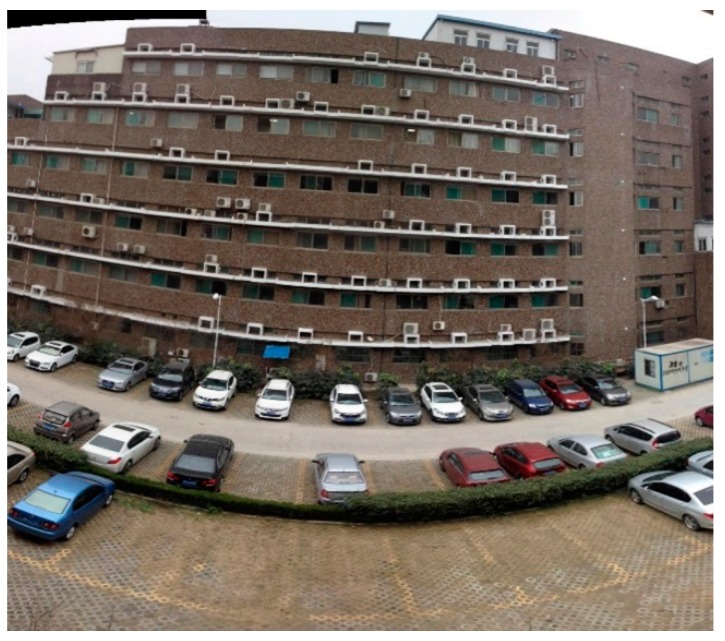
An example of stitched and photometric corrected image obtained with this multi-camera system.

We can obtain large field spherical panorama images as shown in Figure 8. The image is a stitched image with the multi-camera one shot. The distance from the multi-camera system to the building is about 20 m.

## 5. Conclusions and Future Work

A large FOV multi-camera system is calibrated for each sensor and lens. Vignetting and radiometric response of sensors are obtained. We have corrected factors that affect pixel gray values, which cannot objectively reflect the brightness of the scene. The panorama synthesized by using these corrected brightness values can show more realistic landscapes. and the calibration process is an essential step for photometric stereo applications. Multiple sensor systems have higher dynamic range than traditional single sensors with a wide-angle lens. In future work, we will model the geometric properties of cameras and continue to explore the broad prospects for multiple cameras in 3D applications. High-dynamic range information could also be acquired from the overlapping image regions, and tone-mapped or synthetic exposure images can be rendered.

## Figures and Tables

**Table 1:** Result of linear and four order polynomial fitting and evaluation.

Camera Num Cam 1	Coefficients					SSE	R-Square
	P4	P3	P2	P1	P0(*N_dc_*)		
Red (four order)	−1.3 × 10^−5^	0.002	−0.099	5.03	−1.40	24.4	0.9995
(linear)	0	0	0	3.24	7.32	43.1	0.9991
Green(four order)	7.8 × 10^−6^	−8 × 10^−6^	−0.040	5.42	1.39	24.9	0.9990
(linear)	0	0	0	4.12	10.26	51.4	0.9980
Blue(four order)	7.5 × 10^−6^	7.8 × 10^−5^	−0.047	6.12	0.75	30.3	0.9991
(linear)	0	0	0	4.68	10.38	60.2	0.9982

**Table 2:** Ratio of digital number and illuminance with 2/25 ms (1-stop) exposure time and ×1 gain setting.

Camera Num	DN/Lux		
	R	G	B
Cam 1	3.2434	4.1291	4.6884
Cam 2	2.7279	3.5919	4.0800
Cam 3	3.4553	4.4740	4.9788
Cam 4	3.2814	4.1726	4.6718
Cam 5	2.8075	3.6397	4.1231
Cam 6	3.4836	4.3828	4.9772
Cam 7	3.0346	3.9320	4.4479
